# Return to Sport for Professional and Subelite Ice Hockey Players After Arthroscopic Surgery for Femoroacetabular Impingement Syndrome

**DOI:** 10.1177/23259671221089984

**Published:** 2022-05-09

**Authors:** Ida Lindman, Martin Löfskog, Axel Öhlin, Josefin Abrahamsson, Eric Hamrin Senorski, Jon Karlsson, Olufemi R. Ayeni, Mikael Sansone

**Affiliations:** † Department of Orthopaedics, Institute of Clinical Sciences, Sahlgrenska Academy, University of Gothenburg, Gothenburg, Sweden.; ‡ Department of Health and Rehabilitation, Institute of Neuroscience and Physiology, Sahlgrenska Academy, University of Gothenburg, Gothenburg, Sweden.; § Division of Orthopaedic Surgery, McMaster University, Hamilton, Ontario, Canada.; *Investigation performed at Orthocenter/IFK-kliniken, Gothenburg, Sweden, and Department of Orthopaedics, Sahlgrenska Academy, Gothenburg, Sweden*

**Keywords:** femoroacetabular impingement syndrome, FAI, FAIS, return to sport, hip arthroscopy, ice hockey

## Abstract

**Background:**

Femoroacetabular impingement syndrome (FAIS) is a common cause of hip pain, which can prevent ice hockey players from sports participation. Hip arthroscopy is often performed to relieve pain and enable the player to return to sport (RTS) and return to performance (RTP).

**Purpose:**

To determine the RTS and RTP rates for ice hockey players at the professional and subelite levels after hip arthroscopy for FAIS.

**Study Design:**

Case series; Level of evidence, 4.

**Methods:**

High-level ice hockey players who underwent hip arthroscopy for FAIS between 2011 and 2019 were identified using a local hip arthroscopy registry. The player’s level was confirmed with ice hockey–specific web pages and was stratified as subelite or professional. Data on the players’ careers were extracted from these web pages. Player position was divided into goalkeepers, defensemen, and forwards. Data on participation in games included the season before onset of symptoms, the season before surgery, and the first and second seasons after surgery. RTS was defined as returning to ice hockey after surgery, and RTP was considered as returning to the same league at a comparable level to before symptoms.

**Results:**

A total of 80 ice hockey players were included. Comparing presymptom performance with the first season after surgery, the RTS rate was 72%, of which 94% of the players returned to the same or higher level of play. Comparing the presurgery season with the first season after surgery, the RTS rate was 78%. At the second season after surgery, 64% of players still played ice hockey, with a significantly higher return rate among professional players compared with subelite players (96% vs 69%; *P* = .014). Overall, 85% goalkeepers, 74% forwards, and 60% defensemen returned to sport. Only 28% played at least the same number of games during the first season after surgery as they did during the presymptom season.

**Conclusion:**

High-level ice hockey players who underwent hip arthroscopy for FAIS had a high RTS rate, in which the majority returned to the same league. However, only 28% played the same number of games the first season after surgery as they did at the presymptom level. Professional ice hockey players returned more frequently than players on the subelite level.

Hip pain due to femoroacetabular impingement syndrome (FAIS) is frequently reported in ice hockey players.^4,6,12^ FAIS is caused by abnormal morphology in the hip joint, on either the femoral side (cam) or the acetabular side (pincer), which induces chondrolabral injuries in the hip joint.^5^ Ice hockey players have high demands on the hip joint, with repeated movements involving deep flexion, adduction, and internal rotation, which may provoke pain due to FAIS.^7,12^

Hip arthroscopy is regarded as the standard surgical method for treating FAIS, and is designed to reduce pain, increase range of motion, and enable athletes to return to sport (RTS) by correcting the abnormal morphology and restoring normal anatomy of the hip joint.^7,18^ The RTS rate after hip arthroscopy for FAIS has been discussed and has, in general, illustrated good results.^15,20,25^ However, the definition of RTS is often ambiguous, generating widespread outcomes, which complicates pooling of data.^8,19,29^

Moreover, while RTS may generally be high after FAIS surgery, the rates of return to presymptom level and return to performance (RTP) may be lower.^1,8^ A recent consensus meeting from the First World Congress in Sports Physical Therapy in Bern, Switzerland, proposed that RTS should be considered as a continuum from participation to RTP, in which the latter “equals or exceeds pre-symptom level.”^3^ Although RTS for ice hockey players in the US National Hockey League (NHL) has been reported to be >90%,^21,24^ it has been described that NHL players undergoing arthroscopy for FAIS had worse postoperative performance and played fewer games per season compared with matched control players.^9^ The possibility to RTS to a presymptom level is of highest importance for a player at the professional level and one of the main reasons for undergoing surgery.^7^ It has been shown that RTS depends on the level of play and that recreational sportsmen have lower RTS than professional athletes.^15^ It is therefore important to educate the patient preoperatively on the likelihood of RTS and to set realistic expectations.

The aim of this study was to explore the RTS for ice hockey players on the professional and subelite levels by using a clear definition of RTS and RTP. The hypothesis was that there is a high rate of return to ice hockey after arthroscopic treatment for FAIS. Moreover, we aimed to compare RTS at different levels of play and between player positions.

## Methods

### Participants

The local hip arthroscopy registry^23^ was used to identify high-level ice hockey players. This registry includes all arthroscopic hip surgeries performed at 2 hospitals, Orthocenter Gothenburg and Mölndals Hospital/Sahlgrenska University Hospital, and although it is a local registry, the hospitals receive patients nationwide; hence, ice hockey players from throughout Sweden are included in the study. The study inclusion criteria were high-level ice hockey players older than 18 years of age at the time of surgery, who had undergone arthroscopic treatment for FAIS between 2011 and 2019. All surgeries were performed at the 2 hospitals, by 5 surgeons. The definition of “high-level” was playing at either a subelite or professional level, in which professional play comprised the 2 highest leagues in the country playing, such as the NHL, and the subelite level included minor leagues such as college hockey. Ethics committee approval was received for the study protocol.

The collection of ice hockey players was performed in 2 steps. At first, individuals with a self-reported sport of ice hockey registered in the local hip registry and a Hip Sports Activity Scale (HSAS) of 7 or 8 before onset of symptoms were selected. An HSAS of 7 includes players at competitive levels at minor or collegiate leagues, and an HSAS of 8 includes players at the elite level.^17^ To confirm the self-reported level of play and to divide the ice hockey players into subelite and professional levels, performance status was obtained from player websites. Data were retrieved on each players’ professional career from http://www.eliteprospects.com, publicly accessible, and https://hockey.instatscout.com, a closed platform for hockey scouts and agents.

We excluded players who were older than 18 years of age at the time of surgery and players who either had retired before undergoing hip arthroscopy or did not play at a competitive, collegiate, or elite level before the onset of symptoms. The exclusion criteria regarding ice hockey play were confirmed by using the ice hockey–specific web pages. If the patient had a self-reported sport of ice hockey in the hip registry yet was not found in the player websites, this patient was deemed to not have played at a high level and was therefore excluded.

Data were retrieved from the season before symptoms, the season before surgery, and the first and second seasons after arthroscopic treatment. All seasons were considered as the whole consecutive season before and after surgery. The definitions of the seasons are in Table 1. Data collected from the specific hockey websites included player position, stick handedness, number of games played, and level of play. Ice hockey position was divided into defenseman, forward, and goalkeeper. The players were further divided into professional play or subelite level of play depending on which league they were playing in. The hip registry^23^ was queried to extract player data such as age, body mass index, sex, duration of symptoms, side of surgery, diagnosis, and registered sport.

**Table 1:** Definitions of the Included Seasons and Return to Sport

Term	Definition
Presymptom	The entire season before onset of symptoms
Presurgery	The entire season directly before surgery
First season after surgery	The first entire season after surgery
Second season after surgery	The second entire season after surgery
Level of play	Divided into subelite or professional level
Return to sport	Defined as returning to at least 1 game after surgery
Return to performance	Playing at least 85% of the number of games per season played at the presymptom level, at the same or higher league level

### RTS and RTP

We defined RTS as playing at least 1 game of ice hockey at a professional level or subelite level (collegiate or minor league) after surgery. It was classified whether the player had returned to the same, lower, or higher level of play with regard to the league in which they were playing. Level of play was compared between either presymptom or presurgery and first and second seasons after surgery. A player not found playing presymptom was excluded from the analyses regarding comparison of presymptom and postsurgery play. However, for a player found active presymptom, yet not playing the season before surgery, it was considered as an improvement in performance if he was found active in the seasons after surgery when comparing the season presurgery with the season after surgery.

For RTP, each player served as his own control, in which the number of games played per season at the presymptom level was compared with the number of games played per season after surgery. To consider the natural variation in games played between seasons, we performed an analysis of random players, without any known medical history, playing at the same level as the included players in this study. We found that there could be a variation of 15% in games played between seasons. Taking this into account, a player was considered as having RTP if he played at least 85% of the number of games at the same (or higher) league as he did presymptom. The definitions of RTS and RTP are summarized in Table 1.

### Indications for Surgery

All patients were assessed with a physical examination, medical history, and radiographic analysis to determine the diagnosis of FAIS. Ice hockey players with a diagnosis of FAIS and a failed improvement after nonsurgical treatment mainly based on physical therapy and nonsteroidal anti-inflammatory drugs, were eligible for surgery. The surgical treatment has previously been described.^14^

### Statistical Methods

Demographics were described with descriptive statistics as means and standard deviations or as medians and ranges when appropriate. The Fisher exact test was used to evaluate differences between level of play and return to play, and the Mann-Whitney *U* test was used to compare age and symptom duration among players who had returned to sport. All statistical analyses were calculated using SPSS version 27 (IBM Corp). Statistical significance was set as *P* < .05.

## Results

### Player Data

**Figure 1.**
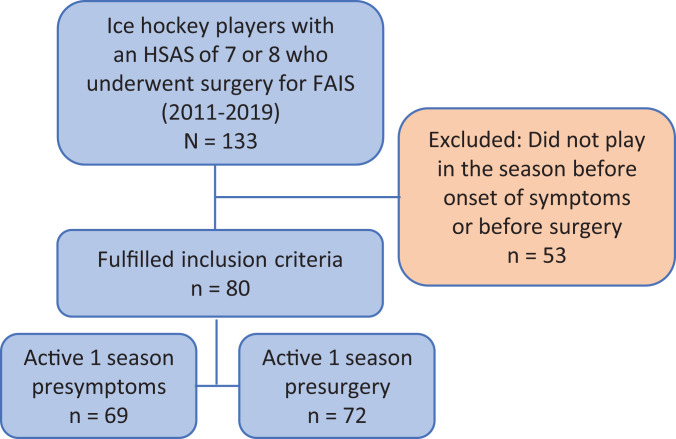
Flowchart of included ice hockey players. FAIS, femoroacetabular impingement syndrome; HSAS, Hip Sports Activity Scale.

A total of 80 high-level ice hockey players fulfilled the inclusion criteria (Figure 1). The average age at the time of surgery was 23 ± 4 years, and start of symptoms was 20 ± 4 years, with a mean symptom duration of 34 ± 22 months. Isolated cam morphology was present in 39% of the players and mixed impingement with both cam and pincer in 61% of the ice hockey players. The characteristics of the players are summarized in Table 2.

**Table 2:** Player Characteristics (N = 80)

Variable	Value
Age, y	23 ± 4 (18-37)
BMI, kg/m^2^	24.7 ± 3.1 (24.8-31.0)
Sex, male	80 (100)
Symptom duration, mo	34 ± 22 (3-120)
Age at start of symptoms, y	20 ± 4 (13-36)
Diagnosis	
Cam	31 (39)
Pincer	0 (0)
Mixed	49 (61)
Position	
Forwards	34 (43)
Defensemen	20 (25)
Goalkeepers	26 (32)
Stick handedness left/right	67/13 (84/16)

^a^ Data are reported as mean ± SD (range) or n (%). BMI, body mass index.

### Return to Sport

**Figure 2.**
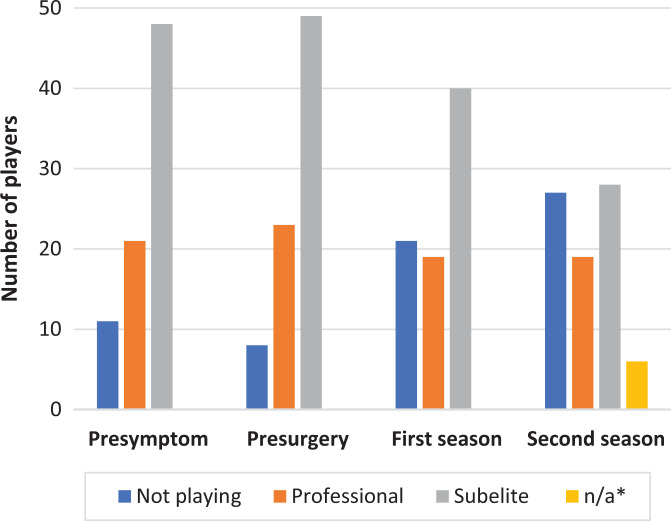
Number of players for each season. n/a, not applicable (*players with recent surgery in which the second season has not yet occurred).

There were 69 ice hockey players with presymptom play, of which 50 (72%) were able to RTS the first season after surgery; 47 of these 50 (94%) returned to the same or higher level of play. Among those who had played in the season presurgery (n = 72), 56 (78%) players returned to sport the first season after surgery. Taken together, 59 of 80 players played the first season after surgery, of which 57 of 59 (97%) returned to the same or higher level of play with regard to league. For the second season after surgery, 6 of the 80 players underwent surgery too recently and their second season had not yet occurred; thus, no data were available for that period, leaving 74 eligible players. Of these, 47 (64%) were still playing ice hockey 2 seasons after surgery (Figure 2).

When comparing different levels of play, 96% (22/23) of the ice hockey players playing in the professional leagues before surgery played the first season after surgery, and 69% (34/49) of players at the subelite level played the first season after surgery. There was a greater RTS among professional ice hockey players compared with subelite players (*P* = .014).

There was no statistically significant difference between positions when comparing RTS at the first season, in which 85% of goalkeepers, 74% of forwards, and 60% of defensemen returned to sport (*P* = .72). There were no significant differences regarding age (*P* = .63) or symptom duration (*P* = .13) in terms of RTS.

### Return to Performance

**Figure 3.**
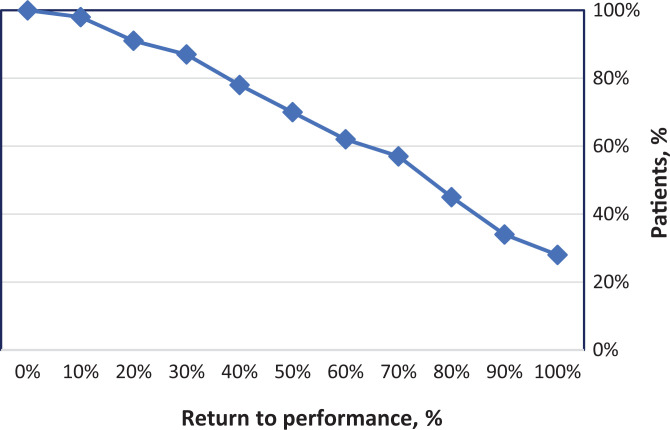
Return to performance compared between numbers of games played presymptom and first season after surgery, described as cumulative percentages.

The mean number of presymptom games played per season was 29 ± 17 (range, 1-68). For active players, regardless of level, the average number of games played the first season after surgery was 23 ± 14 (range, 1-61). The average number of games played the second season after surgery was 26 ± 18 (range, 1-66). During the first season after FAIS surgery, only 13 (28%) were playing the same number, or more, of games as they did presymptom. Based on the definition of RTP as 85% of games played, 37.5% had returned to performance (Figure 3).

## Discussion

The most important finding in this study was that 72% and 78% of active high-level ice hockey players returned to sport during the first season after hip arthroscopy for FAIS compared with presymptom and presurgery levels, respectively. More than 90% of the ice hockey players who had returned to sport the first season after surgery remained on the same level of play. These findings support the theory that active ice hockey players have a high RTS rate, and that it is possible to return to the same level during the first season after surgery.

Although a 78% RTS rate compared with presurgery among ice hockey players should be considered rather high, it is lower than previously reported, in which studies have reported a RTS rate of more than 90% for ice hockey players.^21,24^ However, the additional inclusion of players at the subelite level and not only professional leagues may explain the lower RTS rate reported in this study. There was a significant difference in RTS rates, in which almost all players (96%) at a professional level returned, which is comparable to previous studies. In general, professional players, regardless of sport, RTS to a greater extent than recreational or collegiate players.^2^ Philippon et al^21^ demonstrated that all NHL players included in their study returned to sport within 3.8 months after hip arthroscopic surgery for labral repair. Another study showed that 91% of NHL players returned to sport within 6.8 months after hip arthroscopic surgery.^9^

Motivation and ice hockey as a profession most likely have a strong impact on RTS rates. As players competing at a professional level are dependent on their career and income, they are probably more incentivized to RTS. On the contrary, for a player at subelite level, the choice to RTS may not align with other potential career options, among other factors. In addition to strong personal incentives, professional players may also be affected by extrinsic factors such as support from their teammates and enhanced access to medical professionals, including rehabilitation experts.^11^

It has, however, been debated whether previously described high RTS after hip arthroscopy for FAIS is somewhat optimistic and does not reflect reality.^8,22^ It is well described that RTS rates depend on the definition of RTS used.^3,29^ Previous studies have defined RTS among ice hockey players as returning to skating or participating in games after surgery,^21^ which may overestimate and be confused as RTP, especially with regard to player expectations. In the current study, we used strict definitions of RTS and RTP, resulting in lower results than previously reported. Only 37.5% of the ice hockey players returned to performance, playing at least 85% of the number of games the first season after FAIS surgery as they did presymptom. RTP is an important aspect as there are inherent problems with measuring RTS in FAIS. For an athlete, it is probably important to return to the presymptom level rather than just returning to presymptom play.^3^

Recent studies have demonstrated that ice hockey players play significantly fewer games with fewer starts after hip arthroscopy.^24^ In line with our findings, Ishoi et al^8^ demonstrated that only 30% of their included athletes who had returned to sport reported optimal performance. They used a self-reported assessment of performance in contrast to the definition used in the present study, and when considering participation, only 8% reported as having had optimal full participation.^8^ RTP should be seen as one of the final stages of a continuum of RTS, and the results of this study underpin the value of including such a variable when considering RTS.^3^ Instead of overestimating the RTS rates, a more robust definition of RTP can give both stakeholders and players an honest prognosis on the ability to return to the same performance as presymptom.

There was a trend toward higher RTS rate for goalkeepers compared with defensemen and forwards in the present study. The lack of statistical significance related to this finding may be because of the small number of players in each group (20 goalkeepers). It has been suggested that goalkeepers, because of “butterfly-style” goalkeeping and extreme internal rotation, are more prone to develop FAIS.^10,27,28^ In the current study, 32% of the players were goalkeepers. On a regular hockey team, 10% of players usually constitute goalkeepers. This overrepresentation of goalkeepers has been previously reported,^13^ and the same finding in this study may confirm that they are indeed at higher risk.

Surprisingly, neither symptom duration nor age was associated with lower RTS. It is well known that longer symptom duration is associated with inferior outcomes after FAIS surgery and younger age has previously been associated with higher RTS.^14,16,26^ The reason for the absence of relationship in this study may relate to the sample size of the study population.

There are multiple aspects affecting a player’s choice to RTS and players may retire for other reasons not related to hip pain.^26^ Often, it is difficult to decide if persistent hip pain is the preventive factor for not returning to sport after surgery, and the reason why some athletes do not RTS remains unknown. Future prospective studies are warranted, including presurgical expectations of returning to sport and postsurgical evaluations of limitations to return, including the reason for not returning.

This is a larger ice hockey study with a structured definition of RTS and RTP. The decision to include 2 levels of play (professional and subelite) facilitates the expectation of RTS for the individual player and is thus more generalizable. As the RTS for the professional players may not be generalizable for all athletes, the RTS rate shown for the subelite group may indicate a more realistic outcome for an ice hockey player. Another strength of this study is that the level of play is derived from trustworthy online sources and not self-reported, as in many other previous studies, minimizing the risk of self-reporting bias.

On the other hand, the limitation of using publicly available data increases the risk of observer bias. Yet, this method has been used in previous studies to determine RTS.^9^ Another limitation to this study is the retrospective design, with the inherent limits of such a design. Although no sample size calculation was conducted a priori, all ice hockey players were consecutively included, and the study size is considered large when compared with similar studies. Another limitation is the lack of data on time from surgery to first game played, which could not be found. Contextual factors, such as intention to RTS, were not taken into consideration. Further, symptom duration was self-reported and could be under- or overrated by the athlete. There was no inclusion of intraoperative findings, such as concomitant labral tears or the degree of chondrolabral injury, which potentially could affect the outcome of the surgery and RTS. However, as the aim of the study was to evaluate RTS after hip arthroscopy for FAIS, and not to decide on potential predictors to RTS, this data was not included.

## Conclusion

High-level ice hockey players undergoing hip arthroscopy for FAIS have a high rate of RTS, in which the majority return to the same league. However, only 28% played the same number of games the first season after surgery as they did at their presymptom level. Professional ice hockey players return more frequently than players on the subelite level, 96% vs 69%.
